# Determinants of Social Activity Among Geriatric Patients in Northern Romania: A Cross-Sectional Study

**DOI:** 10.3390/jcm14020565

**Published:** 2025-01-17

**Authors:** Valer Donca, Diana Alecsandra Grad, Marius I. Ungureanu, Constantin Bodolea, Elisabeta Ioana Hirişcău, Lucreţia Avram

**Affiliations:** 1 Department 5—Medical Specicalties, Geriatrics-Gerontology, Faculty of Medicine, Iuliu Hațieganu University of Medicine and Pharmacy Cluj-Napoca, 400012 Cluj-Napoca, Romania; valerdonca@gmail.com (V.D.); avram.lucretia9@gmail.com (L.A.); 2 Department of Public Health, Faculty of Political, Administrative and Communication Sciences, Babes-Bolyai University, 400347 Cluj-Napoca, Romania; m.i.ungureanu@gmail.com; 3 Intensive Care Unit Department, Faculty of Medicine, Iuliu Hațieganu University of Medicine and Pharmacy Cluj-Napoca, 400012 Cluj-Napoca, Romania; c.bodolea@gmail.com; 4 Nursing Department, Iuliu Hațieganu University of Medicine and Pharmacy Cluj-Napoca, 400012 Cluj-Napoca, Romania; ioanahiriscau@gmail.com

**Keywords:** elderly, social activity, public health

## Abstract

**Background/Objectives:** The aging population poses a significant challenge to global public health, impacting the physical, mental, and social health of older adults. Social activity has been widely acknowledged as a protective factor for both mental and physical well-being. Research indicates that consistent engagement in social activities can mitigate the risk of depression, prevent cognitive decline, and support physical functionality. This study aims to explore the correlations and associations between two variables related to social activity (self-reported activity level and time spent with friends) and various other variables among geriatric patients in Northern Romania. **Methods:** This cross-sectional, single-center observational study utilized data from 588 geriatric patients (402 females and 186 males) admitted to the Geriatrics ward of the Municipal Clinical Hospital. The dataset included variables such as sociodemographic information, Geriatric Depression Scale (GDS), Montreal Cognitive Assessment (MoCA), and SARC-F questionnaire scores, time spent with friends, and activity levels. Descriptive statistics were computed alongside statistical tests to examine group differences, associations, and predictive relationships. **Results:** The sample was characterized by variability in age, educational attainment, and pension levels. The statistical analyses revealed significant differences based on education, pension, and civil status. Patients with higher GDS and SARC-F scores had lower odds of spending time with friends or belonging to the active or extremely active groups. Notably, women reported higher GDS scores and lower activity levels compared to men. **Conclusions:** Understanding the factors that influence social activity among older adults is essential for designing targeted interventions aimed at preventing social isolation and fostering healthy aging across diverse demographic and environmental contexts.

## 1. Introduction

The aging population represents a major challenge for global public health, with significant implications for the physical, mental, and social health of older adults. By 2050, the global population of individuals over the age of 60 is projected to reach nearly 2 billion, representing 22% of the world’s population, a demographic shift which imposes significant and growing pressure on healthcare and social protection systems, raising concerns about the capacity of societies to adapt to the functional and structural challenges posed by progressive aging [1,2,3]. In Romania, this process is further exacerbated by socioeconomic and geographic inequalities, disparities between urban and rural areas, limited access to specialized medical resources, and cultural and demographic challenges, all of which significantly contribute to the increase in social isolation among the elderly [4,5,6].

Social activity is recognized as one of the most important protective factors for the mental and physical health of older adults. Studies have shown that consistent social activities help reduce the risk of depression, prevent cognitive decline, and maintain physical functionality. Participation in social activities such as volunteering, community events, or discussion groups fosters a sense of belonging and emotional support, mitigating the negative effects of isolation [2,7]. For example, social activities stimulate cognitive reserve, a concept which describes the brain’s ability to compensate for functional losses by utilizing alternative neural networks developed throughout life [2].

Cognitive reserve is a capacity that is influenced by factors such as educational attainment, participation in social activities, an active lifestyle, and occupational type. Studies suggest that occupations involving intense mental stimulation (e.g., education, research, engineering) can significantly contribute to the development of cognitive reserve. This is attributed to the brain’s adaptability, which is supported by constant exposure to intellectual challenges and complex interactions [8].

However, social isolation remains a significant issue among older adults, particularly in Romania, where the lack of adequate social infrastructure and cultural traditions can limit social interactions, especially in rural areas [9]. Individuals living alone, particularly widowed or unmarried persons, are especially vulnerable to isolation, exposing them to an increased risk of depression, cognitive decline, and other health problems [10]. Intergenerational relationships, more common in rural areas, can provide additional social support but are often insufficient to compensate for the absence of organized social activities [11,12].

Depression and cognitive decline are significant predictive factors in reduced social activity. Depression not only diminishes the desire for social activities but also impairs the ability to form and maintain relationships, while cognitive decline affects communication skills and active participation in community life [2,13]. Similarly, sarcopenia and other physical conditions, such as frailty, limit mobility, reducing opportunities for social interaction [14]. Fear of falling, commonly encountered in this age group, also plays a key role in restricting participation in physical and social activities. This fear often leads to a gradual withdrawal from social life, contributing to increased isolation among older adults [7,10,15].

Sociodemographic factors such as educational attainment, financial resources, and living conditions significantly influence social participation. Individuals with higher levels of education and increased financial resources are more likely to engage in recreational and cultural activities, contributing to better mental health and overall well-being. Conversely, the absence of these resources increases the risk of social isolation and associated health problems [16,17,18].

Understanding the factors related to social activity among older adults is essential for developing effective strategies to prevent social isolation and promote healthy aging. Our study aims to investigate how different variables (sociodemographic as well as those related to mental and physical health) are correlated or associated with social activity among geriatric patients in Northern Romania.

## 2. Materials and Methods

This is a cross-sectional study using data collected retrospectively from the hospital database of patients aged 55+ admitted to the Geriatrics ward at the Municipal Clinical Hospital in Cluj-Napoca, Romania, between 1 January 2023 and 31 January 2024. For this analysis, we included only patients with complete data for the analyzed variables (*n* = 588). We analyzed several sociodemographic variables, such as age, settlement type, education, net pension, civil status, and living conditions. Age, although initially collected as a continuous variable, was transformed for this study into a categorical variable (55–65 years, 66–75 years, 76–85 years, and 86+ years). Settlement type was a binary variable, with the following categories: “urban” and “rural”. Education was a nominal variable with six categories (“no education”, “primary school”, “gymnasium”, “high school”, “university”, and “postgrad”). Net pension, similar to age, was transformed from a continuous into a categorical variable (0–1000 RON, 1001–2000 RON, 2001–3000 RON, 3001–4000 RON, 4001–5000 RON, 5001–6000 RON, and 6001–9000 RON). Civil status was a nominal variable that had four categories (“not married”, “married”, “divorced”, and “widow”). Living conditions had five categories (“house”, “apartment building—elevator”, “apartment building—first level”, “apartment building—no elevator”, and “long-term care facility”).

The first outcome variable was “self-reported activity level”, with three response options of “sedentary”, “active”, and “extremely active”. The second outcome variable was “time spent with friends”, with two response options-“little time” and “much time”. In addition, we also analyzed the scores for three scales: the Geriatric Depression Scale (GDS), the Montreal Cognitive Assessment (MoCA), and the “Strength, assistance with walking, rising from a chair, climbing stairs, and falls” questionnaire (SARC-F).

The Geriatric Depression Scale (GDS) is a commonly used scale to detect depression among geriatric patients. It contains thirty questions (having “yes”/“no” answers), with each question corresponding to a point, depending on the response. Based on the final score, there are three categories: normal (0–9 points), mild depression (10–19 points), and severe depression (20–30 points) [19,20,21].

The Montreal Cognitive Assessment (MoCA) is a validated screening tool that has been used in patients suffering from different pathologies and belonging to different age groups, including geriatric patients, for determining mild cognitive impairment. It has high specificity and sensitivity and has been validated in multiple languages [19,22,23,24,25]. It consists of several tasks that aim to evaluate different cognitive domains. A score below 26 is indicative of cognitive impairment [26].

SARC-F is a screening tool for sarcopenia composed of five questions, one for each domain (“strength, assistance in walking, rising from a chair, climb stairs, falls”), scored with 0 (“none”), 1 (“some”), or 2 (“a lot or unable”). A score of 4 or 4+ can predict sarcopenia [27,28].

For categorical variables, we reported counts and percentages by sex. We used Pearson’s Chi-squared, Kruskal–Wallis, and Fisher’s tests. For the continuous variables, we reported the median, interquartile range (IQR), minimum (min), and maximum values (max) by sex. We used Wilcoxon’s rank-sum and Kruskal–Wallis tests (with post hoc Dunn’s test).

We conducted three binary logistic regressions using “time spent with friends” as a dependent variable and three multinominal logistic regressions using “self-reported activity level” (treated as a nominal variable as the assumptions for ordinal logistic regressions were met only for GDS). In all six regressions, GDS, MoCA, and SARC-F were included as independent variables in the models.

We conducted multiple linear regression using GDS as a dependent variable and sex, education, and pension (for this regression, we used this as a continuous variable, not a range, as it was a better model fit).

We conducted all analyses using R version 4.1.3.

## 3. Results

### 3.1. Descriptive Statistics, Group Comparisons, and Associations

The analyzed sample consisted of 402 female patients and 186 male patients (*n* total = 588). Most patients were aged between 76 and 85 years (female—*n* = 178; male—*n* = 90), listed gymnasium as the last attained educational level (female—*n* = 145; male—*n* = 67), had a pension ranging between 1001 and 2000 RON for female respondents (*n* = 206) and between 2001 and 3000 RON for male respondents (*n* = 71). Regarding living conditions, most patients stated that they were living in a house (female—*n* = 246; male—*n* = 111).

Regarding the self-reported activity level, most female patients rated themselves as sedentary (*n* = 203), and most male patients rated themselves as active (*n* = 101), while for time spent with friends, both male and female patients listed that they spent “little time” with friends (female—*n* = 293; male—*n* = 128).

The median for GDS was 13 for female and 9 for male patients. The median score for MoCA was 21 for female and 22 for male patients and, for SARC-F, 5 for female and 3 for male patients.

Statistically significant differences were found between male and female geriatric patients in terms of SARC-F (W = 46,392, *p*-value < 0.001) and GDS (W = 45,249, *p*-value < 0.001); however, for MoCA, there were not statistically significant differences (W = 35,595, *p* = 0.349).

**Figure 1:**
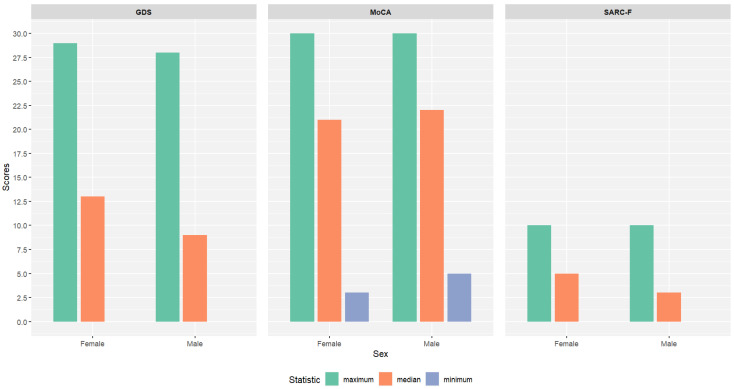
Summary statistics for GDS, MoCA, and SARC-F scores, for 588 geriatric patients (female—*n* = 402; male—*n* = 186). For GDS and SARC-F, the minimum value for both male and female geriatric patients was 0; hence, it does not appear in Figure 1.

**Figure 2:**
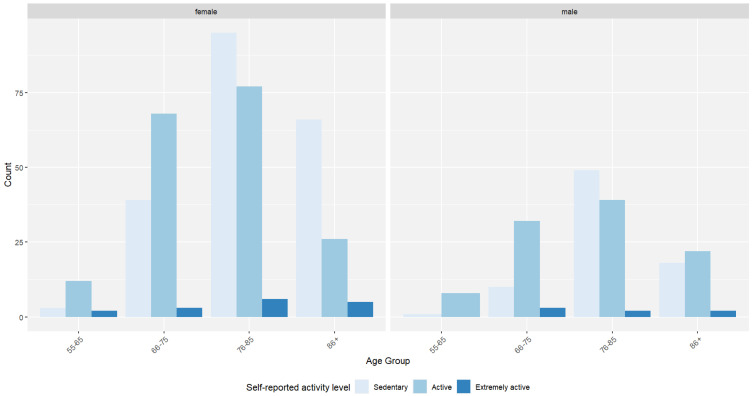
Distribution of self-reported physical activity levels across age groups and sex for 588 geriatric patients (female—*n* = 402; male—*n* = 186).

**Figure 3:**
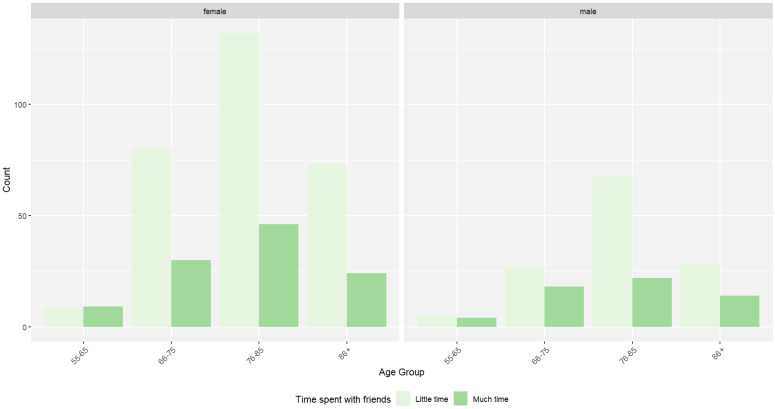
Distribution of time spent with friends across age group and sex, for 588 geriatric patients (female—*n* = 402; male—*n* = 186).

Additional descriptive statistics can be found in Table 1 and are illustrated in Figure 1, Figure 2 and Figure 3.

For categorical variables, statistically significant group differences were reported for education, pension, and civil status.

For the statistically significant categorical variables, we also ran comparisons across education, pension ranges, and civil status in the GDS score. For education, there were differences in the GDS scores across education levels based on the Kruskal–Wallis test results (χ^2^(5) = 27.692, *p* < 0.001). Following the post hoc Dunn test, patients reporting high school as their upper level of education had higher GDS scores compared to patients reporting primary school (*p* = 0.0085), while patients reporting university as the last education level obtained had higher GDS scores compared to patients reporting gymnasium (*p* = 0.028) or primary school (*p* < 0.001). The results for the other pairwise comparisons were statistically significant. Regarding pension ranges, no pairwise comparisons were statistically significant following the Bonferroni correction applied for the post hoc Dunn test. Lastly, for civil status (χ^2^(3) = 21.607, *p* < 0.001), there were higher scores among widowed participants compared to divorced (*p* = 0.019) and married patients (*p* < 0.001).

### 3.2. Results of Linear Regressions

The following linear regressions were conducted between the GDS (dependent variable) and the following combinations: sex and education or sex and pension (as independent variables). For both models, male patients had lower GDS scores than female patients (β = −1.98, *p* < 0.01—first model; β = −1.93, *p* = 0.003—second model); there were no significant effects for education levels, and pension had a negative association with GDS scores, which was marginally significant (β = −0.0005, *p* = 0.053). For GDS combined with sex and civil status, the assumptions needed to conduct a multiple linear regression did not hold.

### 3.3. Results of Binary Logistic Regression

There was a statistically significant association between GDS and time spent with friends (β = −0.09399, SE = 0.01517, and *p* < 0.001). For each one-point increase in the GDS score, patients had 9% lower odds of spending time with friends (OR = 0.91, 95% CI [0.88, 0.94]). Regarding MoCA, there was a statistically significant association between the MoCA scores and the time spent with friends (β = 0.048, SE = 0.016, and *p* = 0.003). For each one-point increase in the MoCA score, patients had 5% increased odds of spending time with friends (OR = 1.05, 95% CI [1.02, 1.08]). Lastly, there was a statistically significant association between the SARC-F scores and the time spent with friends (β = −0.224, SE = 0.037, and *p* < 0.001). For each one-point increase in the SARC-F score, patients had 20% lower odds of spending time with friends (OR = 0.80, 95% CI [0.74, 0.86]).

### 3.4. Results of Multinominal Logistic Regression

The results of the three multinominal logistic regression analyses were statistically significant, with GDS, MoCA, and SARC-F being predictors of the self-reported activity level. For the self-reported activity level, for each one-point increase in the GDS score, patients had lower odds of being in the active (OR = 0.85, 95% CI [0.82, 0.88], and *p* < 0.001) or extremely active categories (OR = 0.81, 95% CI [0.74, 0.88], and *p* < 0.001) compared to the sedentary category. As for the MoCA, for each one-point increase in the MoCA score, patients had significantly higher odds of being in the active (OR = 1.12, 95% CI [1.09, 1.16], and *p* < 0.001) or extremely active categories (OR = 1.08, 95% CI [1.00, 1.17], and *p* = 0.045) compared to the sedentary category. Lastly, for each one-point increase in the SARC-F score, patients had significantly lower odds of being in the active (OR = 0.57, 95% CI [0.52, 0.63], and *p* < 0.001) or extremely active category (OR = 0.56, 95% CI [0.46, 0.69], and *p* < 0.001) compared to the sedentary category.

## 4. Discussion

To the best of our knowledge, this study is the first study to examine the association between the level of social activity and mental health among older adults in Romania.

Our study provides an in-depth examination of the elderly population in northwestern Romania, with a special focus on socioeconomic factors, gender disparities, and health outcomes. The findings highlight several critical aspects that warrant further exploration, emphasizing the interconnected nature of these variables, and are supported by relevant recent works in the literature [29,30].

The analyzed sample demonstrated notable variability in age, educational attainment, and pension income. These findings are supported by specialized studies, which underscore the significant impact of socioeconomic disparities on the health outcomes of elderly populations [30,31]. Education, in particular, has been highlighted as a determinant factor influencing economic resources and access to healthcare services—both critical elements for this demographic group [29,32]. Notably, 62% of participants resided in urban areas, while 38% were from rural regions. This distribution is particularly relevant considering that a large proportion of Romania’s population lives in rural areas. The findings align with research highlighting substantial barriers to healthcare access in rural communities, which may exacerbate health problems among this vulnerable population [31]. Comparative analyses between rural and urban populations reveal that limited resources in rural areas, including inadequate infrastructure and shortages of qualified medical staff, significantly hinder access to medical care and amplify health inequalities.

Educational attainment emerged as a critical factor influencing health outcomes. Since occupational type influences exposure to mentally stimulating activities, future research should include this variable to explore the relationship between professional engagement and social participation among the aging population. Additionally, the literature highlights that occupational type may mediate the effects of economic and educational factors on mental health in older adults. In our study, although sociodemographic and clinical factors such as education and pension were considered, occupational type was not included as a variable [33].

Over half (54%) of the participants had not completed high school, and this educational disparity was reflected in pension levels, with women receiving, on average, significantly lower pensions than men. These observations are consistent with findings from the literature, which highlight the impact of education on economic security [32,34]. Studies emphasize that lower levels of education exacerbate gender disparities in pensions and health outcomes among the elderly [35]. Furthermore, research demonstrates that older women face heightened vulnerabilities, as they not only experience reduced financial security but also report higher levels of depression and cognitive decline. Such evidence underscores the compounded challenges faced by elderly women, warranting targeted interventions [34]. Our results revealed that sedentary participants had significantly higher depression scores, with 50% exhibiting mild-to-moderate depressive symptoms. This finding is supported by research identifying social isolation and lack of physical activity as key predictors of depression.

Furthermore, studies confirm the complex relationship between depression, cognitive decline, and reduced daily activities, highlighting the interconnected nature of these factors [32,36]. The Median Geriatric Depression Scale (GDS) scores were higher for women than men, emphasizing the increased risk of depression among socially isolated individuals, particularly women. Additionally, participants with higher levels of education reported lower depression scores, demonstrating the protective role of education in mental health. This relationship is further supported by research documenting the positive impact of education in facilitating access to robust social networks and resources, which play a pivotal role in preserving mental well-being [37].

Regarding cognitive function, the participants in our study reported median MoCA scores of 21 for women and 22 for men, both below the threshold of 26, indicating cognitive impairment. Active engagement in social and physical activities was associated with higher cognitive scores; each additional point in the MoCA scale increased the likelihood of being physically active by 12%. These findings align with evidence from a study analyzing 677 subjects in China, which highlighted the influence of leisure activities on mental health, emphasizing the mediating role of social support in maintaining cognitive health and delaying decline [31,38].

The association between sarcopenia and physical and social health was particularly pronounced. Sedentary participants had higher SARC-F scores, averaging 5, compared to 3 for active participants. Women exhibited greater vulnerability to muscle mass loss, as reflected by higher scores. These results are supported by studies investigating the association between leisure-time physical activity (LTPA) and sarcopenia among older adults in six countries, which found that participants with low LTPA levels (<150 min/week) had significantly higher chances of developing sarcopenia; this relationship was more pronounced in women [31,39]. Additionally, our analysis showed that each one-point increase in the SARC-F score was associated with a 20% reduction in the likelihood of spending time with friends.

Marital status also had a significant impact on mental health. Widowed participants reported significantly higher GDS scores than married individuals, reflecting the lack of direct social support. These findings align with evidence from recent studies indicating that widowed older adults are at a heightened risk of depressive symptoms due to the absence of close social ties and emotional support networks. Social support has been widely recognized as a protective factor, reducing the prevalence and severity of depressive symptoms by fostering a sense of connectedness and resilience in aging populations [40]. Regarding living conditions, 62.9% of the patients lived in individual houses, a characteristic which may reflect physical isolation, particularly in rural areas where access to resources is limited.

The present study has several limitations that should be acknowledged. The cross-sectional design inherently limits the ability to establish causal relationships, as data were collected at a single point in time. This approach restricts the depth of analysis regarding trends and the progression of mental and social health outcomes among older adults. Future research employing longitudinal designs with multiple follow-up points would offer a more comprehensive understanding of these dynamics, allowing for the exploration of causality and temporal changes. Additionally, the sample comprised exclusively hospitalized patients, which may have disproportionately represented individuals with severe health conditions. This focus limits the generalizability of the findings to the broader elderly population. Including non-hospitalized individuals in future studies would help create a more balanced and representative dataset, thereby offering a clearer and more holistic view of the determinants of social activity in this demographic.

Economic data in the study were limited to pension levels, which, while serving as a useful proxy for financial status, fail to capture the full complexity of economic resources. Household income, savings, or caregiving expenses were not assessed, yet these factors play a critical role in understanding financial strain among the elderly.

For example, studies have demonstrated that higher household income levels are associated with a significant reduction in health risks. Participants in the highest income quintile experienced a 20% reduction in risk compared to those in the lowest income quintile [41]. Furthermore, the interplay between income and social support highlights a critical dynamic: while all lower income quintiles (Q1–Q4) showed significantly higher average depression scores compared to the highest quintile (Q5), higher social support levels mitigated these effects, particularly in the lowest income group. In the first quintile, for instance, high social support was associated with significantly reduced depression scores, illustrating the protective role of social networks for economically disadvantaged individuals [42].

The absence of data on participants’ occupational history constitutes another limitation. Information on the type of job, years of employment, or exposure to cognitively stimulating activities was not collected, despite evidence suggesting that occupational factors significantly influence cognitive reserve and, consequently, mental health and social engagement in older adults. Incorporating such data in future research would provide deeper insights into how lifelong engagement in various professions impacts the aging process.

Another significant limitation lies in the exclusion of polypharmacy from the analysis. Medications, including antidepressants, are prevalent in geriatric populations and can profoundly affect mental health and social activity, both positively and negatively. Exploring the role of polypharmacy in future research would yield valuable insights into its implications for quality of life and social integration among the elderly.

Finally, the dataset lacked detailed information on the frequency, duration, and quality of social interactions. These dimensions are essential for understanding the nuanced relationship between social engagement and psychological well-being. Future studies should prioritize the inclusion of these factors to provide a more holistic and multidimensional analysis.

Despite these limitations, this study offers valuable insights into the determinants of social activity among geriatric patients in Northern Romania. By identifying critical areas for intervention and exploration, it lays the groundwork for future research and policy efforts aimed at improving the well-being of this vulnerable population. Policy recommendations include developing educational programs, improving access to healthcare services in rural areas, and initiating programs promoting mental health through social and physical activities. Tailoring these interventions to the specific needs of older adults across diverse settings could significantly reduce inequalities and enhance the quality of life for this demographic.

## 5. Conclusions

The findings underscore the intricate interplay between social activity, mental health, and physical well-being among elderly individuals, emphasizing the need for holistic, tailored interventions which address the multifaceted challenges of aging. By fostering accessible social infrastructures, promoting cognitive and physical engagement, and addressing socioeconomic disparities, we can pave the way toward healthier, more fulfilling aging trajectories for Romania’s geriatric population and beyond.

## Figures and Tables

**Table 1:** Descriptive statistics for categorical variables reported by sex.

Variable	Female	Male	
Age			0.755
55–65 years	17 (2.89%)	9 (1.53%)	
66–75 years	110 (18.71%)	45 (7.65%)	
76–85 years	178 (30.27%)	90 (15.31%)	
86+ years	97 (16.5%)	42 (7.14%)	
Settlement type			0.7043
Urban	236 (40.14%)	113 (19.22%)	
Rural	166 (28.23%)	73 (12.41%)	
Education			0.0005
No education	4 (0.68%)	2 (0.34%)	
Primary school	83 (14.12%)	23 (3.91%)	
Gymnasium	145 (24.66%)	67 (11.39%)	
High school	126 (21.43%)	56 (9.52%)	
University	41 (6.97%)	37 (6.29%)	
Postgrad	3 (0.51%)	1 (0.17%)	
Pension			*p* < 0.0005
0–1000 RON	9 (1.53%)	4 (0.68%)	
1001–2000 RON	206 (35.03%)	35 (5.95%)	
2001–3000 RON	113 (19.22%)	71 (12.07%)	
3001–4000 RON	52 (8.84%)	43 (7.31%)	
4001–5000 RON	15 (2.55%)	14 (2.38%)	
5001–6000 RON	5 (0.85%)	10 (1.70%)	
6001–9000 RON	2 (0.34%)	9 (1.53%)	
Civil status			*p* < 0.0001
Not married	6 (1.02%)	8 (1.36%)	
Married	111 (18.88%)	107 (18.2%)	
Divorced	18 (3.06%)	7 (1.19%)	
Widow(er)	267 (45.41%)	64 (10.88%)	
Living conditions			0.6254
House	246 (41.84%)	111 (18.88%)	
Apartment building—elevator	52 (8.84%)	21 (3.57%)	
Apartment building—first level	23 (3.91%)	11 (1.87%)	
Apartment building—no elevator	78 (13.27%)	39 (6.63%)	
Long-term care facility	3 (0.51%)	4 (0.68%)	
Self-reported activity level			0.1357
Sedentary	203 (34.52%)	78 (13.27%)	
Active	183 (31.12%)	101 (17.18%)	
Extremely active	16 (2.72%)	7 (1.19%)	
Time spent with friends			0.3581
Little time	293 (49.83%)	128 (21.77%)	
Much time	109 (18.54%)	58 (9.86%)	

## Data Availability

The dataset used during the current study is available from the corresponding authors upon reasonable request.
